# The *Streptococcus pneumoniae* *yefM-yoeB* and *relBE* Toxin-Antitoxin Operons Participate in Oxidative Stress and Biofilm Formation

**DOI:** 10.3390/toxins10090378

**Published:** 2018-09-18

**Authors:** Wai Ting Chan, Mirian Domenech, Inmaculada Moreno-Córdoba, Verónica Navarro-Martínez, Concha Nieto, Miriam Moscoso, Ernesto García, Manuel Espinosa

**Affiliations:** 1 i-DNA Biotechnology (M) Sdn Bhd. A-1-6 Pusat Perdagangan Kuchai, No. 2, Jalan 1/127, Kuchai Entrepreneurs Park, Kuala Lumpur 58200, Malaysia; chanyting@hotmail.com; 2 Centro de Investigaciones Biológicas, Consejo Superior de Investigaciones Científicas, 28040 Madrid, Spain; miriandomenechlucas@hotmail.com (M.D.); inmamoreno395@gmail.com (I.M.-C.); veronica.vnm@outlook.es (V.N.-M.); cnieto@cib.csic.es (C.N.); Mirian.Moscoso.Naya@sergas.es (M.M.); 3 CIBER de Enfermedades Respiratorias (CIBERES), Instituto de Salud Carlos III, 28040 Madrid, Spain

**Keywords:** *Streptococcus pneumonia*, toxin–antitoxin, *relBE*, *yefM-yoeB*, oxidative stress, biofilm formation

## Abstract

Type II (proteic) toxin-antitoxin systems (TAs) are widely distributed among bacteria and archaea. They are generally organized as operons integrated by two genes, the first encoding the antitoxin that binds to its cognate toxin to generate a harmless protein–protein complex. Under stress conditions, the unstable antitoxin is degraded by host proteases, releasing the toxin to achieve its toxic effect. In the Gram-positive pathogen *Streptococcus pneumoniae* we have characterized four TAs: *pezAT*, *relBE*, *yefM-yoeB*, and *phD-doc*, although the latter is missing in strain R6. We have assessed the role of the two *yefM-yoeB* and *relBE* systems encoded by *S. pneumoniae* R6 by construction of isogenic strains lacking one or two of the operons, and by complementation assays. We have analyzed the phenotypes of the wild type and mutants in terms of cell growth, response to environmental stress, and ability to generate biofilms. Compared to the wild-type, the mutants exhibited lower resistance to oxidative stress. Further, strains deleted in *yefM-yoeB* and the double mutant lacking *yefM-yoeB* and *relBE* exhibited a significant reduction in their ability for biofilm formation. Complementation assays showed that defective phenotypes were restored to wild type levels. We conclude that these two loci may play a relevant role in these aspects of the *S. pneumoniae* lifestyle and contribute to the bacterial colonization of new niches.

## 1. Introduction

*Streptococcus pneumoniae* (the pneumococcus) is a Gram-positive, microaerophilic human pathogenic bacterium which colonizes the human nasopharynx of nearly 75% of humans [1], and is the causal agent of community-acquired pneumonia. This is an acute infection that causes the death of nearly 1.5 million children under the age of five. Other less severe pneumococcal infections may be 10 times more frequent, with an average stay in hospitals of 4 to 5 days. Thus, *S. pneumoniae* infections represent a heavy burden not only because of their high toll on human lives but also because of their economic impact in terms of health expenses [2]. Furthermore, *S. pneumoniae* is naturally transformable, which may have led to the selection of the hyper-recombinogenic features of the bacterium [3]. Appearance of pneumococcal resistance to antibiotics as well as selection of serotypes to which there is no vaccine yet [4,5], have led to the search of different strategies to deal with pneumococcal infections [6]. Among novel approaches, the use of the toxic proteins encoded by type II toxin-antitoxin (TA) systems as potential antimicrobials have been proposed [6,7,8,9,10,11]. Type II TA systems are, in general, organized as an operon in which the gene encoding an unstable antitoxin precedes the gene encoding a stable toxin. Under steady-state conditions, the cognate antitoxin neutralizes the toxin, generating a harmless protein–protein TA complex that auto-regulates their synthesis by binding to regions encompassing the promoter of the operon [12]. Under stressful circumstances, however, TA synthesis is triggered, and the antitoxin is degraded more rapidly leading to the release of the toxin that subsequently exerts its toxic effects in the cell.

Among type II pneumococcal TAs, data mining from 48 sequenced pneumococcal genomes (assembled or in contigs) led to the prediction of the existence of up to 10 putative TAs in some strains, and yet this number could be an underestimation [13]. Validation of these results led to the discovery of a fourth *bona fide* operon encoding a PhD-Doc type II TA system, whereas some of the predicted pneumococcal TAs did not behave like classical ones, i.e., the putative toxin was not neutralized by overexpression of the putative cognate antitoxin [14,15]. The rest of the pneumococcal type-II TAs have been functionally studied in some detail, namely *pezAT* [16,17,18], *relBE2Spn* [19,20], and *yefM-yoeB* [21,22]. Another pneumococcal *relBE* homolog, termed *relBE1Spn*, was shown to not be functional [15], corroborating previous observations that over-expression of the putative pneumococcal RelE1*Spn* toxin did not result in RNA cleavage [23]. Thus, it was proposed to consider *relBE1Spn* as an evolutionary remnant and to rename *relBE2Spn* as *relBE*, which is the only functional *relBE* homolog in the pneumococcus [15]. Overproduction of the pneumococcal RelE or YoeB toxins led to cell growth arrest, and arrested cells could be rescued by overproduction of the cognate antitoxins. Both pneumococcal toxins cleave translating mRNA like their *Escherichia coli* counterparts [23,24]. Analyses of ~100 pneumococcal clinical isolates together with database-mined strains showed high conservation of the *relBE* locus, whereas the *yefM-yoeB* operon was not present in several strains [13,25]. In the case of toxin PezT, its overproduction in *E. coli* led to growth arrest, but cell growth was resumed without the need of a concomitant synthesis of the PezA antitoxin [16]; such growth profile was similar to the one reported for *E. coli* cells overproducing the Zeta toxin (a homolog of toxin PezT) of plasmid pSM19035 [26]. PezT toxin inhibited bacterial cell wall synthesis by phosphorylating UDP-*N*-acetylglucosamine to UDP-*N*-acetylglucosamine-3′-phosphate in the presence of ATP and Mg^2+^; the phosphorylated UDP-*N*-acetylglucosamine inhibits the MurA enzyme which, in turn, is essential in the initial stages of peptidoglycan biosynthesis. Thus, free PezT provoked cell autolysis [17] and, concomitantly, deletion of the *pezAT* operon led to pneumococcal cells being more resistant to β-lactam antibiotics and exhibiting increased transformability [18].

It was proposed that the role of the pneumococcal TAs in the bacterium lifestyle could be related to processes such as biofilm formation and response to stressful conditions [27]. To test these possibilities we have constructed isogenic *S. pneumoniae* R6 strain-derivatives lacking *yefM-yoeB*, *relBE*, and both operons, and complemented the deficiency by cloning either operon in a stably inherited plasmid. Bacterial growth, environmental stresses, and biofilm formation were tested with these strains. Cells lacking *yefM-yoeB*, *relBE*, or both operons were more sensitive to oxidative stress than the wild type (*wt*), although no differences were found when the strains were stressed by pH or by the presence of Zn^2+^ cations. Biofilm formation was significantly impaired in cells lacking *yefM-yoeB* but could be reversed by complementation. It also was slightly not significantly, reduced in strains lacking *relBE*, and furthermore was reduced up to ~50% in the strain lacking both operons. 

## 2. Results

### 2.1. Genetic Organization of the Pneumococcal Operons yefM-yoeB and relBE 

**Figure 1:**
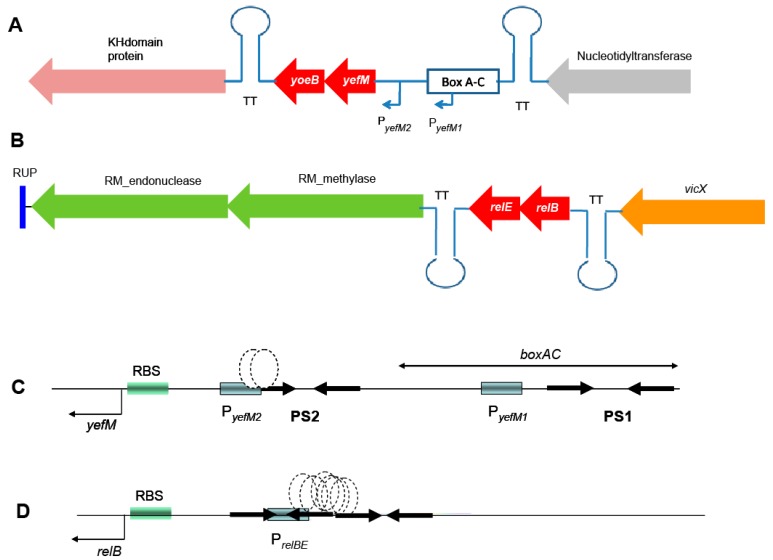
Genetic organization of the chromosome of *S. pneumoniae* R6 surrounding the two TAs studied here (depicted in red). (**A**): The *yefM-yoeB* operon, in which relevant genes in the region encode a nucleotidyl transferase (grey), and a KH-domain protein (pink). (**B**): The *relBE* operon in this strain is surrounded by *vicX* (a metal-dependent hydrolase, orange) and a type II restriction/modification endonuclease (green). Both *yefM-yoeB* and *relBE* operons are flanked by transcription terminators (TT). (**C**): Expanded view of the control region of *yefM-yoeB*, indicating the *boxAC* element, the two promoters of *yefM-yoeB* (boxed), the palindromic sequences (PS1 and PS2, arrows), the binding site of the YefM::YoeB protein complex (stippled ovals), and the ribosome binding site (RBS). (**D**): The control region of the *relBE* operon with the same symbols as in (**C**).

The pneumococcal *yefM-yoeB* operon is located at coordinates 1566450 to 1567001 in the chromosome of R6, a nonencapsulated strain derived from the capsular type 2 clinical isolate strain D39 [28]. Differently from the other three pneumococcal TAs, the surrounding genes of *yefM-yoeB* do not seem to include any mobile DNA and its genetic structure is conserved among the different strains that encode this TA operon (Figure 1A; [13,15]). The operon is flanked by a nucleotidyl transferase and a putative protein with a KH-domain [29], respectively (Figure 1A). In the case of *relBE*, we found a considerable variability among different pneumococcal isolates, and up to six different organizations around the operon, four of them being the most abundant (Supplementary Figure S1; [13,25]). The one present in strain R6 (coordinates 1105118 to 1104862; [28]) showed that the *relBE* operon is located between gene *vicX* (a metal-dependent hydrolase) and a type II restriction/modification endonuclease (Figure 1B). Both *yefB-yoeB* and *relBE* operons are flanked by transcription terminators, suggesting that they are self-contained units.

Concerning the transcriptional control regions, the *yefM-yoeB* operon seems to be unique for type II TAs in the sense that it is transcribed from two promoters, one of them not self-regulated by YefM. The promoter placed proximal to the *yefM* ATG initiation codon (P*_yefM2_*) has a long palindromic sequence encompassing its –35 region. This palindrome sequence (PS2 in Figure 1C) was shown to be the binding site of the YefM-YoeB proteins by gel retardation and footprinting assays, and it is also involved in the auto-regulation of the operon [21]. The second promoter (P*_yefM1_*) is provided by the insertion of a pneumococcal BOX element upstream of it and is not regulated by either YefM or the YefM-YoeB protein complex [21]. This organization could lead to a higher basal level of transcription for the *yefM*-*yoeB* operon. If this is the case, then the strains carrying the *boxAC* elements (around 20%) would have the ability to cope faster with sudden changes in their environment [12,13,21]. In the case of *relBE*, a single promoter placed in a region with two inverted repeats directs the synthesis of both genes (Figure 1D). This region also harbors the DNA binding site(s) of the RelB::RelE protein complex [19]. 

**Figure 2:**
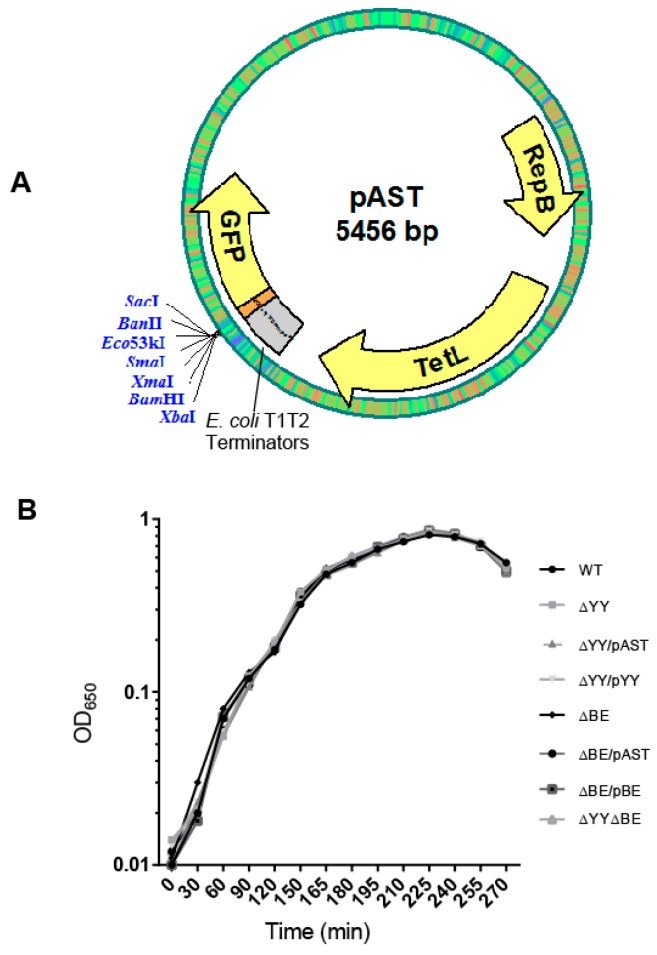
(**A**) The plasmid vector used to clone the pneumococcal operons used for complementation assays. Plasmid pAST [34] is based on the pMV158 replicon [35]; it harbors the T1T2 transcriptional terminators from *E. coli* [36] and a multiple cloning site to fuse, if desired, any gene to the gene encoding the bacterial-optimized version of the Green Fluorescent Protein [37]. The entire *yefM-yoeB* or *relBE* operons with its own transcription/translation signals were inserted between the BamHI and SacI restriction sites. (**B**) Growth curves of the strains used in this work.

Given the possible involvement of TA systems in the pneumococcal lifestyle [13,15], we were interested in testing the behavior of TA mutants of strain R6 lacking either *yefM-yoeB*, *relBE* or both, when confronted with environmental stress. As in the case of *pezAT*, we performed these analyses in the pneumococcal host [18], rather than using an unrelated host like *E. coli*, as done before in our and in other labs [22,30]. Through gene replacement strategies [31,32], the pneumococcal *yefM-yoeB* operon was replaced by a Km^R^ cassette, whereas *relBE* was deleted and replaced by a Cm^R^ module [33]. A double mutant, defective in both operons was also constructed. Plasmid vectors carrying either operon (under their own transcription/translation signals) were constructed for complementation assays (Figure 2A), although a doubly-complemented strain could not be attained (see Methods). Thus, the isogenic strains derived from R6*wt* were: (i) single deletion mutants Δ*yefMyoeB* (ΔYY) and Δ*relBE* (ΔBE); (ii) the double mutant Δ*yefM-yoeB,* Δ*relBE* (ΔYYΔBE); (iii) the single mutants ΔYY or ΔBE harboring the control (‘empty’) plasmid pAST (ΔYY/pAST and ΔBE/pAST, respectively), and (iv) the single mutants harboring the complementing plasmids pASTYY and pASTBE (ΔYY/pTYY and ΔBE/pBE, respectively).

We proposed that the role of the two R6 RNA interferases in the pneumococcus lifestyle could be related to bi-stable behaviors [13], such as (i) phase variation in the morphology of the colonies, due to the presence of pneumococcal BOX elements [38]; (ii) response to stressful environments [25], or (iii) biofilm formation as a way to survive under adverse conditions. In the case of the pneumococcal *pezAT*-TA genes, deletion of the entire operon made cells more resistant to lysis than the R6*wt* strain but the capacity of biofilm formation was not altered [18]. It was then interesting to analyze first the growth rates of *wt* and mutant strains as well as those harboring plasmids. No significant differences in the growth rate of the cultures were found between the strains (Figure 2B).

### 2.2. Deletion of the Pneumococcal Operons Reduce Response to Oxidative Stress

**Figure 3:**
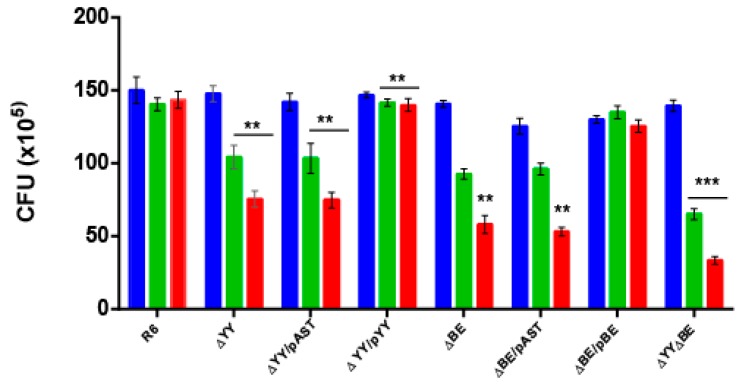
Deletion of the *yefM-yoeB* or the *relBE* operons leads to reduced resistance to oxidative stress. The eight pneumococcal isogenic strains were grown in AGCH medium at 37 °C, to OD_650_ = 0.30. The cells of each culture were centrifuged, washed, and concentrated twice in PBS; 50 µL samples were untreated (blue) or treated with an equal volume of H_2_O_2_ to give a final concentration of either 5 mM (green) or 10 mM (red) H_2_O_2_. Incubation proceeded at 37 °C, 20 min, and serial dilutions were plated on AGCH agar and incubated at 37 °C. The number of CFU was determined, and the results were represented as total CFU numbers. The assays were repeated five times and the mean (±SD) were determined. The asterisks denote significance value of *p* < 0.01 (**) and *p* < 0.001 (***).

Response to stressful conditions is one of the main roles in which TAs are involved [13,39,40,41,42,43,44]. When *S. pneumoniae* colonizes a new niche, like the lungs, one important hurdle that the bacterium encounters is the presence of free reactive oxygen species (ROS). Thus, hydrogen peroxide (H_2_O_2_) released from the human cells is a key element to be overcome by the invading bacteria [45,46]. Pneumococci are able to produce high levels of H_2_O_2_ when growing aerobically, probably to outcompete other bacteria sharing their niche, but *S. pneumoniae* do not synthesize catalase to break the peroxide [47]. To determine whether the two studied operons could be related to this specific stress, we subjected the pneumococcal cells to sub-inhibitory concentrations of H_2_O_2_ attempting to imitate the environment during infection [48]. After several trials, we chose 5 and 10 mM H_2_O_2_ as concentrations in which a small reduction, if any (~95%) in the colony-forming unit (CFU) counts in the *wt* strain were found. All the strains were subjected to H_2_O_2_ treatment and the CFU at the end of the incubation period were determined. The results (Figure 3) showed: (i) a significant reduction in the CFU of strains ΔYY, ΔBE, or in the same cells harboring the ‘empty’ plasmid when compared to the *wt* cells; at 10 mM H_2_O_2_, the values obtained were *wt* = 143.4 ± 2.64 (100%), ΔYY = 75.4 ± 2.56 (52.6%) and ΔBE = 58.01 ± 2.70 (40.4%); (ii) reduction in the CFU counts were fully restored in the complemented strains; and iii) reduction in the CFU was more pronounced in the double mutant, around 25% of the CFU of the R6*wt* strain at 10 mM H_2_O_2_. We conclude that one of the roles of both TA operons is to protect, at least partially, the pneumococcus from oxidative stress and this, in turn, points that these operons might play a role in bacterial infection, at least under certain conditions. A mechanism linking response to oxidative stress and induction of persistence in *Mycobacterium bovis* was reported [49] where a modification of the tRNA anticodons for threonine or leucine leads to the activation of translation of proteins related to the response to ROS and, ultimately, to the induction of persistence [49].

To know whether these two pneumococcal operons could also participate in the response to other stress-inducers, we tested the response to low pH and elevated concentrations of Zn^2+^. Acidic response in *S. pneumoniae* seems to be regulated by ComE through a competence-stimulating peptide-independent pathway, leading to cell lysis [50] and to the release of virulence factors [51]. Zinc ions are found in different concentrations depending upon the tissue, ranging from 5 µM in the nasopharynx to 300 µM in the lungs [52,53]. The results showed that none of the pneumococcal cells were able to grow at the lowest pH tested, and that 1 mM or 0.5 mM Zn^2+^ was highly toxic to all strains tested. No significant differences between the strains were observed (Supplementary Figure S2).

### 2.3. Pneumococcal Cells Lacking the RNA-Interferase Operons Have Reduced Biofilm Formation

**Figure 4:**
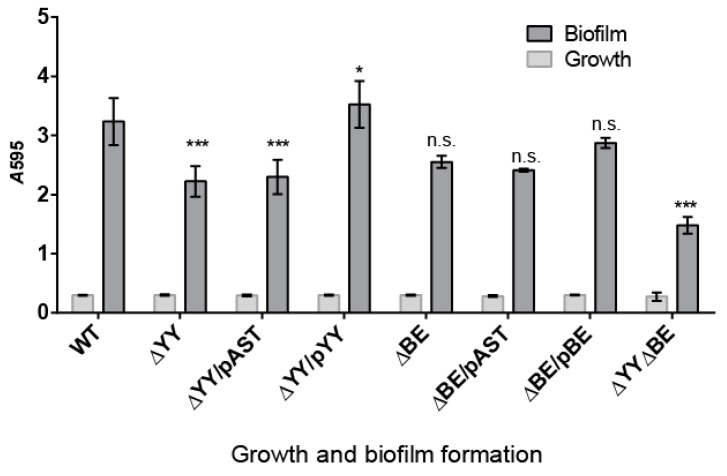
Deletion of either *yefM-yoeB* or the two *yefM-yoeB* and *relBE* operons significantly diminishes pneumococcal biofilm formation. Biofilm formation was determined by the ability of cells to adhere to the walls and base of 96-well (flatbottom) polystyrene microtiter dishes using a modification of a previously reported protocol [62]. Unless stated otherwise, cells grown in C+Y medium to an OD_550_ of ~0.5–0.6 were sedimented by centrifugation, resuspended in an equal volume of the indicated prewarmed medium, diluted 1/100, and then dispensed at a concentration of 200 μL per well. Plates were incubated at 34 °C, for 6 h, and the biofilm formed was stained with 0.2% crystal violet [62]. Bars show the growth (light grey bars) and biofilm formation (dark grey bars) of the indicated strains. The values of biofilm formation were normalized for absorbance. The results were repeated 9 times for biofilms and 19 times for cell growth. The *p* values were 0.001 (***) and 0.05 (*); n.s., not significant.

*S. pneumoniae* infections may adopt a biofilm way of growth, thus protecting the bacterial community from antibiotic treatments, turning many of pneumococcal infections into chronic ones [54]. In addition, some of the type II TAs found in *E. coli* participate in persistence and in biofilm formation [55,56,57,58], and this later feature is one of the few clearly confirmed TA functions [59]. Thus, we decided to test whether deletion of the two pneumococcal operons showed their participation in biofilm formation. The biofilm forming capacity of *S. pneumoniae* R6*wt* and its derivatives constructed here were analyzed by growing the strains at 34 °C (the temperature of the human nasopharynx [60]). After 6 h incubation, bacterial growth was determined by measuring the *A*_595_, and the biofilm formed was stained with crystal violet and rinsed to remove non-adherent (planktonic) cells [61]. The bacterial growth was similar in all the strains, and robust biofilm formation was detected and quantified (Figure 4). Comparison of the biofilm formation between the *wt* and the ∆YY mutant showed statistically significant differences, which were not significant enough for the ∆BE mutant. The calculated percentage of biofilm formation was reduced from the *wt* (3.235 ± 0.397) to ~70% both in the ∆YY (2.224 ± 0.258), and in the ∆YY/pAST (2.298 ± 0.291) strains, with a *p* < 0.001, indicative of values with high significance. These biofilm values were restored to the *wt* levels, or even slightly higher, in the ∆YY/pYY (3.524 ± 0.258) complemented strain (Figure 4), and this latter value was also significant (*p* < 0.05). In the case of the strain defective in the *relBE* operon, differences were also found; however, we could not assign significant values to the reduction observed. However, substantial reduction in biofilm formation (~45%) was observed for the double mutant: from the *wt* (3.235 ± 0.397) to the mutant (1.481 ± 0.14) values (*p* < 0.001). Therefore, deletion of *yefM-yoeB*, and much more deletion of both *yefM-yoeB* and *relBE* operons led to a substantial defect in pneumococcal biofilm formation. Since there was a further decrease in the double mutant as compared to the ∆YY mutant, it would appear that the role played by the *relBE* TA could be compensated for by *yefM-yoeB*.

**Figure 5:**
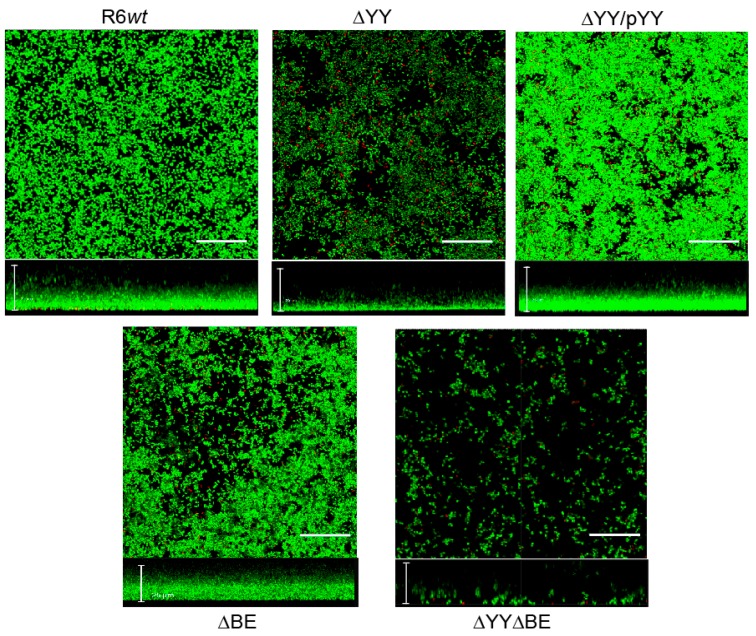
Influence of the deletion of the *yefM-yoeB* and/or *relBE* operons on the viability of biofilm-growth of *S. pneumoniae* as determined by CLSM. Cells in the biofilms were stained with the *Bac*Light LIVE/DEAD kit to reveal viable (green fluorescence) and non-viable (red fluorescence) bacteria. Horizontal and vertical three-dimensional reconstructions of 40 (*x*–*y* plane) or 68 scans (*x*–*z* plane) are shown. In all images the scale bar is 25 µm.

We next took images of the biofilms of strains R6*wt*, ∆YY, ∆YY/pYY, ∆BE, and ∆YY∆BE using confocal laser scanning microscopy (CLSM) (Figure 5). The results clearly showed that deletion of the *yefM-yoeB* operon led to a substantial decrease in the thickness of the biofilm and to an increased proportion of dying cells (judged by the many red cells in the preparations) during biofilm formation compared to the parental strain. These defects were fully complemented in the strain ∆YY/pYY. Further, the strain harboring the double mutation exhibited poor ability of biofilm formation with an increase in dead cells and a substantial decrease in its thickness.

## 3. Discussion

We have studied the two TA systems of strain R6 of *S. pneumoniae* encoding two RNA interferases, namely *yefM-yoeB* and *relBE* in the cognate host by constructing sets of isogenic strains defective in one of the two operons or in both of them. No differences in the growth rate of the strains were found (Figure 2B), and two effects could be observed in the deleted strains: (i) reduction in the survival to H_2_O_2_ treatments (Figure 3), and (ii) a decrease in the ability for biofilm formation (Figure 4 and Figure 5). Our results show that the two TAs encoded by *S. pneumoniae* strain R6 play a similar and cooperative role in oxidative stress response and biofilm formation, thereby contributing to the pneumococcal lifestyle. These two TAs exhibited a different genetic organization and their participation in the pneumococcal lifestyle seems to be similar. However, the additive effects found in the double mutant point to different roles, and epistasis of *yefM-yoeB* over *relBE* in biofilm formation was evident (Figure 4). The differences between both TAs may lay in their regulation: There is a low but constitutive synthesis of *yefM*-*yoeB* from the unregulated promoter P*_yefM1_* (Figure 1C; [21]), whereas transcription of *relBE* is repressed by the RelB::RelE protein complex [12,19,20]. Biofilm formation in pneumococcus is affected by environmental conditions, especially when the cells colonize niches different from their usual habitat [60,61]. However, and to our knowledge, there is no information on the degradation of the pneumococcal antitoxins by proteases; a detailed analysis of the role of each partner (toxin or antitoxin) in the phenotypes observed here is still needed. 

Participation of the type II-TA *mqsRA* (motility quorum-sensing regulator) on biofilm formation in *E. coli* was reported and it was shown that toxin MqsR was increased by biofilm formation and that its architecture was affected by stimulating cell motility [63]. Further, antitoxin MqsA was shown to participate not only in biofilm formation but also in the regulation of the general stress responses, such as oxidative stress [42,64]. Upon oxidative stress, the antitoxin MqsA is degraded by Lon and ClpXP proteases, and, as a result, the stringent response is triggered, and the bacterial state changed from planktonic to sessile (biofilm) [64]. In the case of the widespread *mazEF* TA system [65,66], it was recently shown that the antitoxin MazE from *Pseudomonas aeruginosa* participates in biofilm formation, rather than the toxin MazF [67]. Further, genes responsive to stress are triggered during colonization, and they also participate in biofilm formation which, in turn, is influenced by multiple TA systems (extensively reviewed in References [57,68]).

Taking all the above results together, we believe that the two pneumococcal operons would participate actively in the pneumococcal lifestyle at least in two ways. Firstly, the presence of the operon would protect the cells from the H_2_O_2_ released from their eukaryotic host and thereby potentially contributing to its virulence. The pneumococcal strain R6 encodes the Rgg transcriptional regulator that operates on a regulatory network of genes, some of them related to resistance to H_2_O_2_; deletion of this regulator leads to increased sensitivity to oxidative stress and to a reduction in biofilm formation [48]. Whether there is a relationship between Rgg and the genes encoding the two pneumococcal TAs studied here remains unknown, although the Clp protease, known to cleave antitoxins, appeared to be regulated by Rgg [48,69]. Secondly, reduction in the biofilm formation in the strains defective in the two operons would indicate that those cells would be either more prone to dispersal from biofilms than the *wt* [70], or more liable to lose viability under biofilm conditions. If we consider the former case, then cells lacking these operons would have higher possibilities of success than the *wt* in the colonization of new niches because of a higher rate in the release of planktonic cells. As in the case of the *pezAT* operon [18], a delicate equilibrium between pneumococcal cells having or not having these two TAs seems to exist, although the scenario would be different. In the case of *pezAT*, loss of functions (resistance to β-lactams and genetic competence) and gain of other traits (virulence) associated to the presence/absence of this TA were found [18]. In the present case, we can speculate that the strains that harbor the two operons would have more chances of success in the colonization of eukaryotic niches that release H_2_O_2_, whereas cells lacking the operons would be more prone to detachment from biofilms. It would be most interesting to repeat the experiments performed here but using a variety of pneumococcal strains [13,25] and to perform a detailed study on the involvement of these TAs on the pneumococcal virulence and colonization ability.

## 4. Materials and Methods

### 4.1. Culture Conditions, Plasmids, and Construction of Bacterial Strains

*S. pneumoniae* R6, a nonencapsulated strain derivative from D39 strain (serotype 2), was used throughout the study [71], as well as the isogenic derivatives made in this study (below). Pneumococcal cells were grown at 37 °C in microaerophilic conditions, using one of the three following media: (i) AGCH supplemented with 0.2% yeast extract and 0.3% sucrose; (ii) the same medium supplemented with 3% agar (to give a final concentration of 1.5% agar), or (iii) 0.08% yeast extract (C+Y medium [34,72,73]. Growth curves were monitored by OD_650_ readings every 30 min on a Bausch & Lomb spectrophotometer; the assays were repeated three independent times and the mean and standard deviation (SD) were calculated.

Plasmids used to construct the isogenic complementing strains were pJS3, a replicon derivative of plasmid pLS1 [72] harboring a *cat* gene that confers resistance to chloramphenicol (Cm^R^; [33]), and plasmid pR410 which contains a *kan* gene (kanamycin resistance, Km^R^; [74]). Plasmid pAST [34] was used as the vector to construct the complementing plasmids. When selection was required, cultures were grown in the presence of Km (250 µg/mL), Cm (1.5 µg/mL), or tetracycline (Tc, 1 µg/mL). Genomic DNA from *S. pneumoniae* was prepared by standard protocols [31].

The set of isogenic strains were constructed as follows (schematized in Supplementary Figure S3; [31,32]):

- *S. pneumoniae* Δ*yefM-yoeB* (herein referred to as ΔYY): the entire *yefM-yoeB* operon was replaced with the *kan* gene cassette from pR410. This gene (1053 bp) was amplified using primer pair kmF/kmR, whereas regions flanking *yefM-yoeB* upstream fragment (581 bp) and downstream fragment (583 bp) were amplified using primer pairs L-FyefMyoeB/L-RyefMyoeB and R-FyefMyoeB/R-RyefMyoeB, respectively (Table 1). Equimolecular amounts of the three PCR products (1053, 581 and 583 bp) were amplified by using primers L-FyefMyoeB and R-RyefMyoeB to obtain a linear 2174 bp-fused product containing the *kan* gene delimited by the flanking regions of the *yefM-yoeB* genes.

*- S. pneumoniaeΔrelBE* (termed ΔBE): the *cat* gene from pJS3 (1048 bp) was PCR-amplified using primers CmF and CmR (Table 1). The primer pairs L-FrelBE/L-RrelBE and R-FrelBE/R-RrelBE were used to amplify the left and right flanking regions, respectively, of the *relBE* operon, obtaining two products of 611, and 607 bp, respectively. Primer L-RrelBE contains complementary sequences to the promoter region of the *relBE* operon and to the 5′ region of the *cat* gene. The primer R-FrelBE contains complementary sequences to the 3′ region of *relE* and to the 3′ region of the *cat* gene. Primers L-FrelBE and R-RrelBE include complementary sequences to the 5′ and 3′ regions, respectively, flanking the *relBE* operon. Equimolecular amounts of the three PCR products (1048, 611 and 607 bp) were amplified by using primers L-FrelBE and R-RrelBE to obtain a linear fused product containing the *cat* gene delimited by the flanking regions of the *relB* and *relE* genes. After generating the desired DNA substrates, the fused products were used to transform into *S. pneumoniae* strain R6 as reported [18], and transformants were selected on AGCH-agar plates containing Km or Cm for ΔYY or ΔBE strains, respectively. Five colonies from each construction were selected and the presence of the gene replacement construct was checked by direct sequencing of the entire chromosomal DNA region, or by sequencing an amplicon of this region (Secugen, Centro de Investigaciones Biológicas-CSIC, Madrid, Spain).

- *S. pneumoniae* Δ*yefM-yoeB,* *ΔrelBE* (termed ΔYYΔBE) lacking both operons was constructed by transformation of the ΔBE mutant (Cm^R^) with chromosomal DNA prepared from the ΔYY mutant (Km^R^), followed by selection with both antibiotics. The mutations of the selected transformants were re-confirmed by sequencing of PCR products of the desired regions (Secugen, CIB, Madrid, Spain), using primers L-FrelBE/L-RrelBE or L-FyefMyoeB/L-RyefMyoeB, and both, combined with the primer pairs relB2P/relE2c, or Sec 1.1/Sec 2.1, respectively (Table 1).

Complementation assays were done by constructing strains harboring either plasmid pASTYY (carrying the *yefM-yoeB* operon) or plasmid pASTBE (carrying the *relBE* operon). Both pneumococcal operons were cloned under their own transcriptional and translational signals into the multicloning site of plasmid pAST (5456 bp; [34]). This vector carries the T1T2 transcriptional terminators from *E. coli* that ensure no transcriptional read-through from the *tetL* gene that could affect the expression of the cloned genes. Plasmid pASTYY was constructed by cloning a 798 bp DNA fragment that was amplified by the use of primers yefMyoeB-F, and yefMyoeB-R (Table 1). In the case of plasmid pASTBE, a 644 bp chromosomal DNA fragment was amplified by the use of primers relBE-F and relBE-R (Table 1). 

The cloned operons were thus flanked by BamHI (at 5′) and SacI (at 3′) restriction sites, respectively. Plasmids were rescued by transformation of strain R6*wt*, and then transferred to strain ΔYY or ΔBE, respectively. Several transformants from each construct were selected. The genomic DNAs were extracted with Bacterial Genomic DNA Isolation Kit (Norgen Biotek Corp, Thorold, ON, Canada), whereas plasmid DNA was extracted with High Pure Plasmid Isolation kit (Roche). The protocols were slightly modified to account for the low G+C content of the pneumococcal genome and the constructs were verified by sequencing of the entire region. The possibility of gene conversion from mutant to *wt* genotypes [31] was discarded by PCR detection of the chromosomal regions of the selected strains. We were unable to construct a double mutant strain harboring the two plasmids because of incompatibility. Attempts at cloning both operons in the same plasmid, or cloning the operons into different compatible plasmids and establishing them into the recipient *S. pneumoniae* Δ*yefM-yoeB,* Δ*relBE* also failed. 

### 4.2. Oxidative Stress Assays

Oxidative stress assays were done as reported [18,48] with the following modifications: The pneumococcal strains were grown in the AGCH medium as described before [34] until the middle exponential phase (OD_650_ = 0.3; ~2 × 10^8^ cells/mL). For each strain, 1 mL of cells was centrifuged and suspended in 500 µL of PBS (pH 7.0), and 50 µL were mixed with an equal volume of H_2_O_2_ (Merck, Kenilworth, NJ, USA), to give a final concentration of either 5 mM or 10 mM H_2_O_2_; the control mixtures contained cells and PBS. Incubation proceeded at 37 °C for 20 min, and serial dilutions were plated on AGCH agar and incubated at 37 °C. The number of CFU was determined, and the results were represented as the percent of survival relative to the control. These assays were repeated five independent times and the mean ± SD and significance were calculated.

### 4.3. Biofilm Formation and Quantification

Biofilm formation assays were performed as previously described [61,62]. Pneumococcal strains were grown in C+Y medium to OD_550_ of 0.5–0.6. Cells were sedimented by centrifugation and resuspended and diluted 1:100 in C medium. Diluted cells were dispensed (~4.5 × 10^6^ CFU per well) in 96-well polystyrene microtiter dishes (Costar 3595, Corning Inc., Corning, NY, USA). After 6 h of incubation at 34 °C, the biofilm formed was stained with 0.2% crystal violet and rinsed to remove non-adherent (planktonic) bacteria. After solubilizing the biofilm (sessile bacteria) in 95% ethanol, the absorbance (*A*_595_) was determined using a plate reader (VERSAmax microplate absorbance reader, Molecular Devices, San José, CA, USA). The CFU of planktonic and sessile bacteria was ~2.5 × 10^8^ and ~1.5 × 10^8^ CFU/mL, respectively. The results represent the mean ± SD and significance of three independent experiments, each of them carried out in triplicate.

### 4.4. Confocal Laser Scanning Microscopy (CLSM)

For observation of biofilms by CLSM we omitted the strains that did not exhibit significant differences in biofilm formation. Cells were grown in C medium on glass-bottom dishes (WillCo-dish, WillCo Wells B. V., Amsterdam, The Netherlands) for 5 h at 34 °C. The biofilm was rinsed to remove non-adherent bacteria and then stained with the LIVE/DEAD *Bac*Light bacterial viability kit L-13152 (Invitrogen-Molecular Probes, Carlsbad, CA, USA) for monitoring the viability of bacterial populations as a function of the membrane integrity of the cell [61]. The staining procedure involved incubation for 15 min at room temperature in the dark. The biofilms were observed at 63× magnifications using a Leica TCS-SP2-AOBS-UV confocal laser-scanning microscope equipped with an argon ion laser. The excitation/emission maxima for the dyes were approximately 480/500 nm (SYTO 9) and 490/635 nm (propidium iodide). Image acquisition was performed by using LCS software from Leica (Version 2.0, Laica Microsystems, Heidelberg, Germany, 2001), and projections were obtained through the *x–y* plane (individual scans at 0.5-µm intervals) and *x–z* plane (images at 3-µm intervals).

### 4.5. Statistical and Computer Analyses

All experiments were independently performed at least in triplicate. Experimental results were calculated and plotted with the aid of the GraphPad Prism software (version 6.0, GraphPad Software, La Jolla, CA, USA, 2012). All values are expressed as mean (±SD). Data from oxidative stress and biofilm assays were subjected to normality tests and analyzed using two-tailed Student’s *t*-test with *p* values < 0.05 that were considered as significant. Significances are indicated with asterisks: *** *p* < 0.001, ** *p* < 0.01, * *p* < 0.05, and n.s., not significant. Figures were processed with GraphPad Prism and Adobe Illustrator CS6 (Adobe Systems, San José, CA, USA, 2012).

## Figures and Tables

**Table 1:** Primers used in this work.

Primers	Sequences 5′ → 3′	Restriction Sites *
kan-F	AGCAGAGCTCCTTATCGATACCGTCGACCTC	SacI
kan-R	AGCAACTAGTCCCCTATCTAGCGAACTTTTA	SpeI
CmF	CGGATTTTATGACCGATGATG	-
CmR	TAACGCGGCAGGTTAGTGAC	-
KmF	CTTATCGATACCGTCGACCTC	-
KmR	CCCCTATCTAGCGAACTTTTAG	-
L-F yefMyoeB	TTTCTTAGAACGTTTTATGCCTTC	-
L-R yefMyoeB	GAGGTCGACGGTATCGATAAGCGCGATTTGAATTTGATTTTCG	-
R-F yefMyoeB	CTAAAAGTTCGCTAGATAGGGGGTCTACTGTAAAGTAGGCTTTTTC	-
R-R yefMyoeB	CTCGTCAAATTGTCGTCCTT	-
L-F relBE	ATGAAAAGACCTGGCAAGCTATG	-
L-R relBE	CATCATCGGTCATAAAATCCGTATAAAAAGAACACCTTCTCAGCG	-
R-F relBE	GTCACTAACCTGCCCCGTTAGGTCATCGGAGAGATATTTATTGA	-
R-R relBE	GACTTCATCTGAAACCTCACG	-
Sec 1.1	CAAACTAAGTCTACTGAAAGGTAGG	-
Sec 2.1	TGTTTTTACCTCATTTTATTGTTATTCC	-
yefMyoeB-F	CAGAGGATCCTCTACTGAAAGGTAGGCTTT	BamHI
yefMyoeB-R	CAGCGAGCTCAAATAGTAGTTTAGTAGAGA	SacI
relBE-F	CAGAGGATCCAAAGAACGCTGAGAAGGTGT	BamHI
relBE-R	CAGCGAGCTCGTAAGCCCTATTATATCATA	SacI

* Restriction sites introduced are underlined.

## Supplementary Materials

The Supplementary Material for this article can be found online at: http://www.mdpi.com/2072-6651/10/9/378/s1. Figure S1: Polymorphisms in the pneumococcal *relBE* operon. Figure S2: Effect of pH- and Zn^2+^-induced stresses on pneumococcal growth. Figure S3: Construction of mutant strains by gene replacement.

## Abbreviations

bp	base pair(s)
CFU	colony-forming units
CLSM	Confocal Laser Scanning Microscopy
nt	nucleotide(s)
OD	optical density
TA	Toxin-Antitoxin
*wt*	wild type
