# Supplementary material for: The Streptococcus pneumoniae yefM-yoeB and relBE Toxin-Antitoxin Operons Participate in Oxidative Stress and Biofilm Formation
Source: Toxins (Basel). 2018 Sep 18;10(9):378. doi: 10.3390/toxins10090378 (PMC6162744; doi:10.3390/toxins10090378)
Supplement: Supplementary file 1 [file toxins-10-00378-s001.pdf]

Supplementary Materials: The *Streptococcus pneumoniae yefM-yoeB* and *relBE* Toxin-Antitoxin Operons Participate in Oxidative Stress and Biofilm Formation

Wai Ting Chan, Mirian Domenech, Inmaculada Moreno-Córdoba, Verónica Navarro-Martínez, Concha Nieto, Miriam Moscoso, Ernesto García and Manuel Espinosa

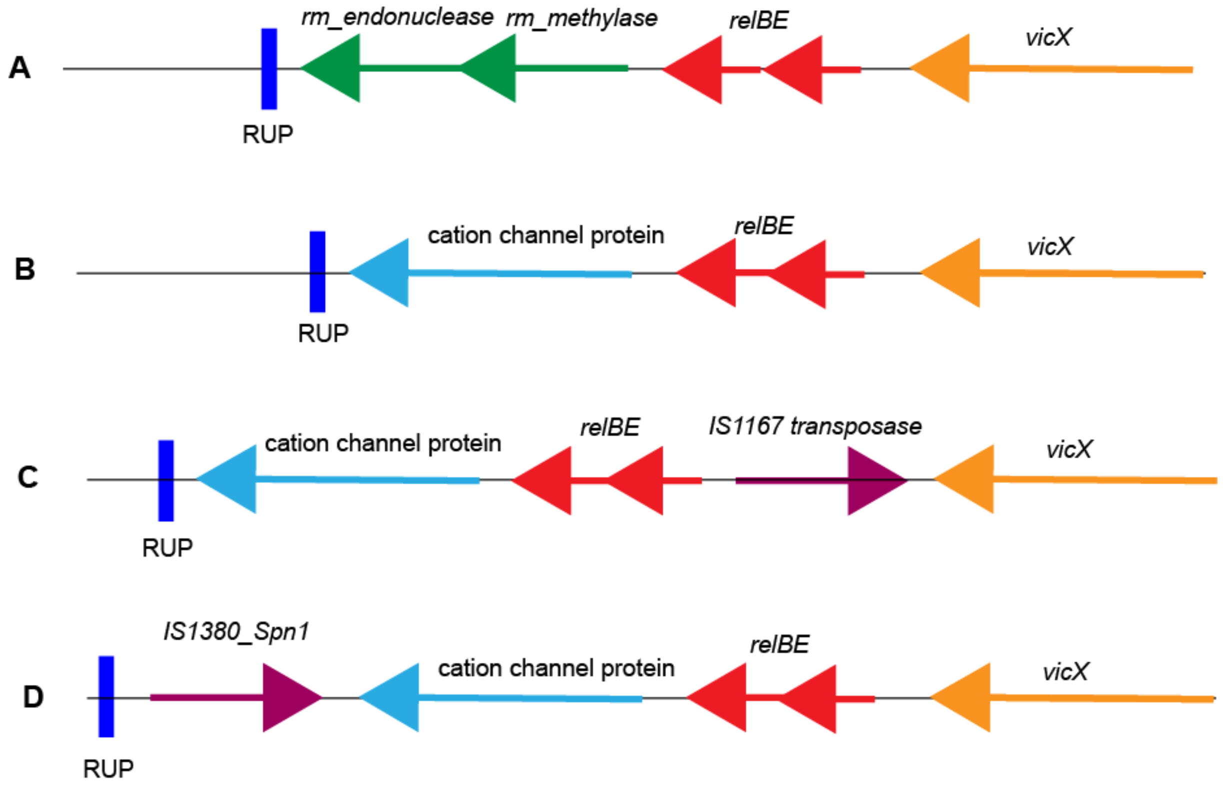

Figure S1. Polymorphisms in the pneumococcal *relBE* operon. In general, *relBE* genes are flanked by gene *vicX* (a metal-dependent hydrolase) and a RUP (**r**epeat **u**nit of **p**neumococcus) element. (**A**) Genes encoding a restriction-modification methylase and type II restriction endonuclease; (**B**) A gene encoding a potassium_cation channel protein; (**C**) same as in B) but with a gene encoding an IS*1167* transposase in the opposite orientation; (**D**) same as in (**B**) but with an IS*1380*-SpnI element in opposite orientation of the gene encoding the cation channel protein. The most frequent variations are depicted, although other organizations have been reported [1,2].

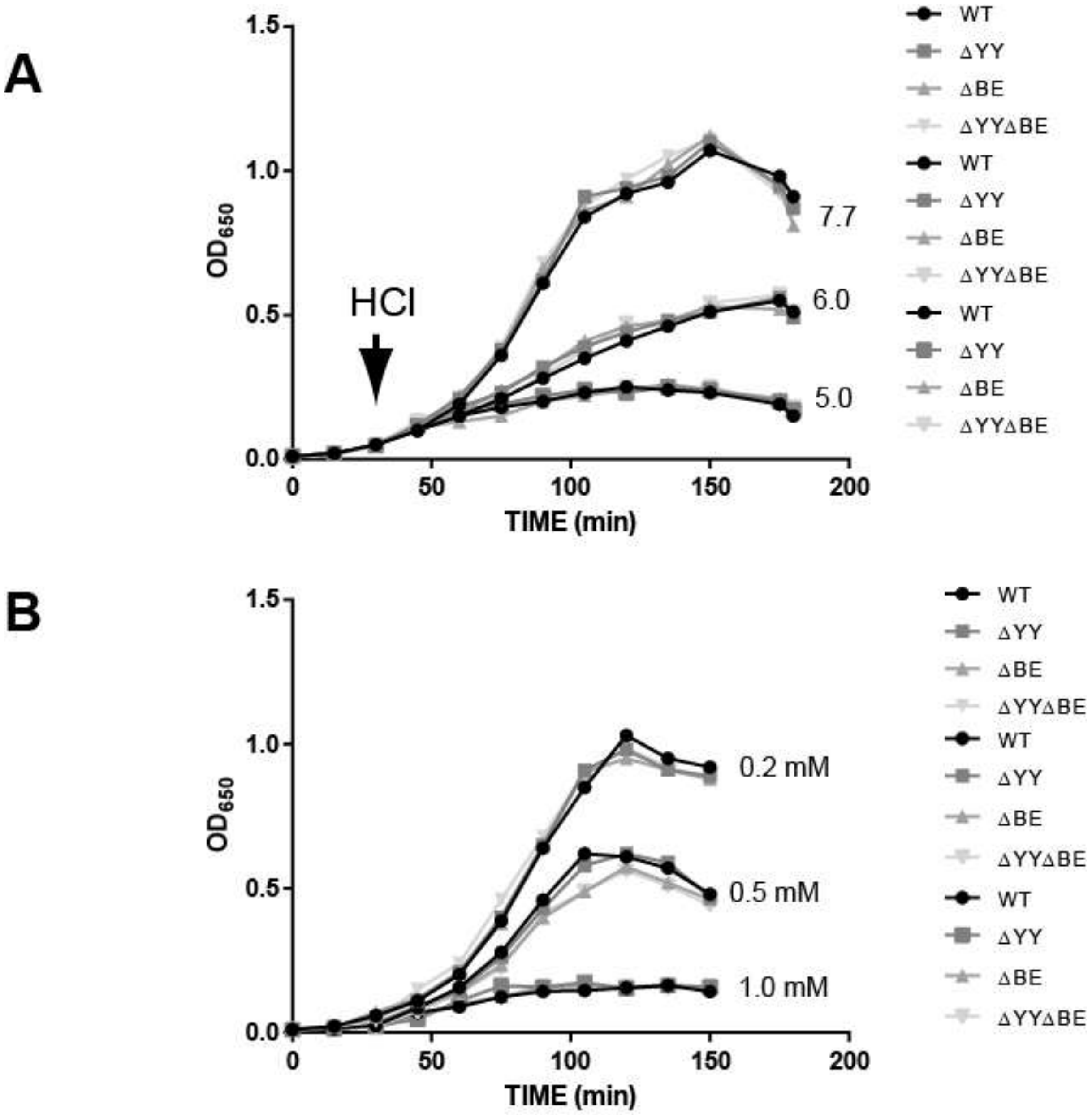

Figure S2: Effect of pH- and Zn^{2+}-induced stresses on pneumococcal growth. The indicated strains were subjected to the stresses caused by low pH or by elevated concentrations of Zn^{2+}. In all cases, cells were grown in AGCH medium, pH 7.7, at 37 °C in microtest tissue culture plates (Becton Dickinson, Franklin Lakes, NJ, USA) and incubated in a plate incubator Multiskan Ascent (Thermo Scientific, Waltham, MA, USA) to measure the OD_{650}. (**A**) In the acidic stress experiments, cells were grown to OD_{650} = 0.15 (~10^8 CFU/mL) and the cultures were divided into three; 0.5 N HCl (vertical arrow) was added to two of them to adjust the medium to pH 6.0 and 5.0, whereas the pH of the third culture was kept at pH 7.7 (the pH of the AGCH medium). (**B**) In the Zn^{2+}-caused stress, the cells were grown in AGCH medium, pH 7.7, supplemented with the indicated concentrations of ZnSO4.

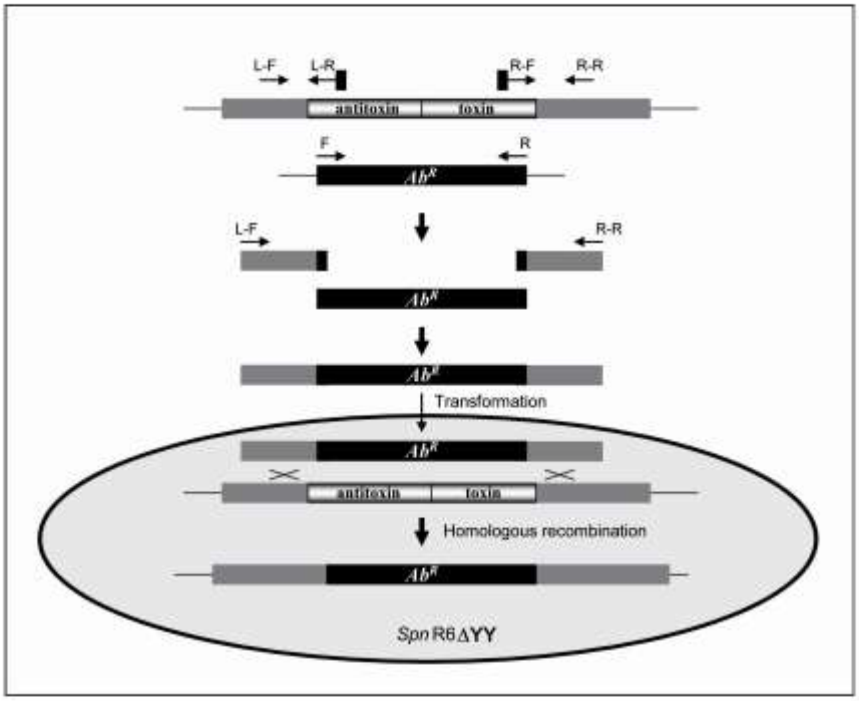

Figure S3. Construction of mutant strains by gene replacement. A first PCR step amplified the antibiotic resistance cassette (Km^R for *yefM-yoeB*, or Cm^R in the case of *relBE*) and the left and right flanking regions of the TA operons. In the second PCR step, mixtures of equimolecular amounts of the three products were used to obtain fused products containing the antibiotic resistance cassette delimited by the flanking regions of the antitoxin and toxin genes. These DNA products were used to transform *S. pneumoniae* R6*wt*. By homologous recombination, the antibiotic resistance cassette replaced the antitoxin and toxin genes in the chromosome to construct strains ΔYY and ΔBE.

References

1. Chan, W.T.; Moreno-Córdoba, I.; Yeo, C.C.; Espinosa, M. Toxin-antitoxin genes of the Gram-positive pathogen *Streptococcus pneumoniae*: so few and yet so many. *Microbiol. Mol. Biol. Rev.* **2012**, 76, 773–791.
2. Nieto, C.; Sadowy, E.; de la Campa, A.G.; Hryniewicz, W.; Espinosa, M. The *relBE2Spn* toxin-antitoxin system of *Streptococcus pneumoniae*: role in antibiotic tolerance and functional conservation in clinical isolates. *PLoS ONE* **2010**, 5, e11289.
